# The use of fire to preserve biodiversity under novel fire regimes

**DOI:** 10.1098/rstb.2023.0449

**Published:** 2025-04-17

**Authors:** Roger Puig-Gironès, Marina Palmero-Iniesta, Paulo M. Fernandes, Imma Oliveras Menor, Davide Ascoli, Luke T. Kelly, Tristan Charles-Dominique, Adrian Regos, Sandy Harrison, Dolors Armenteras, Lluís Brotons, Sergio de-Miguel, Gian Luca Spadoni, Rachel Carmenta, Manoela Machado, Adrian Cardil, Xavier Santos, Maitane Erdozain, Guillem Canaleta, Christian Niel Berlinck, Quel Vilalta-Clapés, Florent Mouillot, Michele Salis, Marcello Verdinelli, Valentina Bacciu, Pere Pons

**Affiliations:** ^1^ Universitat de Girona Departament de Ciencies Ambientals, Girona, Catalunya, Spain; ^2^ Universitat de Barcelona Departament de Biologia Evolutiva Ecologia i Ciencies Ambientals, Barcelona, Spain; ^3^ Universidade de Tras-os-Montes e Alto Douro, Vila Real, Portugal; ^4^ AMAP (Botanique et Modélisation de l'Architecture des Plantes et des Végétations), Institut de Recherche pour le Développement Centre de Montpellier, Montpellier, Occitanie, France; ^5^ Environmental Change Institute, School of Geography and the Environment, University of Oxford, Oxford, UK; ^6^ University of Torino, Turin, Italy; ^7^ University of Melbourne, Melbourne, UK; ^8^ Institute of Ecology and Environmental Sciences, Paris, France; ^9^ Department of Community Diversity and Ecosystem Functioning, Sorbonne University, Paris, France; ^10^ Forest Science and Technology Centre of Catalonia, Solsona, Spain; ^11^ Geography and Environmental Science, University of Reading, Reading, UK; ^12^ Universidad Nacional de Colombia, Bogotá, Colombia; ^13^ CREAF, Bellaterra, Catalunya Spain; ^14^ CSIC, Cerdanyola del Vallès, Catalunya Spain; ^15^ Department of Agricultural and Forest Sciences and Engineering, University of Lleida, Lleida, Spain; ^16^ AMAP, Montpellier, Occitanie, France; ^17^ Department of Agriculture, Forest and Food Science; University of Torino, Torino, Italy; ^18^ Department of Science, Technology and Society; University School for Advanced Studies IUSS Pavia, Pavia, Italy; ^19^ Tyndall Centre for Climate Change Research and School of Global Development, University of East Anglia, Norwich, UK; ^20^ School of Geography and the Environment, University of Oxford, Oxford, UK; ^21^ Woodwell Climate Research Center, Falmouth, MA USA; ^22^ CIBIO, Centro de Investigação em Biodiversidade e Recursos Genéticos, Universidade do Porto, Porto, Portugal; ^23^ Pau Costa Foundation, Taradell, Spain; ^24^ Centro Nacional de Pesquisa e Conservação de Mamíferos Carnívoros, Instituto Chico Mendes de Conservação da Biodiversidade, Atibaia, Brazil; ^25^ CEFE, Montpellier, Languedoc-Roussillon, France; ^26^ Institute of BioEconomy National Research Council Sassari Branch, Sassari, Sardegna, Italy

**Keywords:** biodiversity conservation, decision-making practices, human-driven fire, integrated fire management, prescribed burning, wildfire

## Abstract

Novel fire regimes are emerging worldwide and pose substantial challenges to biodiversity conservation. Addressing these challenges and mitigating their impacts on biodiversity will require developing a wide range of fire management practices. In this paper, we leverage research across taxa, ecosystems and continents to highlight strategies for applying fire knowledge in biodiversity conservation. First, we define novel fire regimes and outline different fire management practices in contemporary landscapes from different parts of the world. Next, we synthesize recent research on fire use and biodiversity, and provide a decision-making framework for biodiversity conservation under novel fire regimes. We recommend that fire management strategies for preserving biodiversity should consider both social and ecological factors, iterative learning informed by effective monitoring, and developing and testing new management actions. An integrated approach to learning about fire and biodiversity will help to navigate the complexities of novel fire regimes and preserve biodiversity in a rapidly changing world.

This article is part of the theme issue ‘Novel fire regimes under climate changes and human influences: impacts, ecosystem responses and feedbacks’.

## 1. Introduction

Humans have long used fire to shape environments and meet societal needs [1,2]. For thousands of years, this has included manipulating the timing and location of ignitions and the amount and distribution of fuels [3]. More recently, human actions have also started to modify biotic distributions and climate patterns at global scales, influencing fuels and their availability to burn. These changes are already causing shifts in terrestrial ecosystems and contributing to biodiversity declines globally [4].

Fire regimes reflect patterns of recurrent fires and can be characterized by properties such as frequency, intensity, severity, spatial pattern and season [5,6]. Fire regime properties vary among ecosystems; for example, some tropical grasslands experience annual fires, while boreal forests might not experience fires for centuries. There is growing evidence that humans are altering the historical range and variation of fire patterns [7], and inducing environments different from those that humanity has experienced [8,9]. Deviations from historical fire patterns—which we refer to as novel fire regimes—can include increased or reduced fire activity [10,11]. While the causes and consequences of novel fire regimes are contentious, there is a consensus that multiple drivers are in play, including climate change, land use, biotic mixing and their underlying societal causes [12].

Understanding fire regimes and how they are changing is important because they modulate biodiversity across scales—from species to whole ecosystems [13,14]. In forests and woodlands with frequent high-intensity fires, plants have evolved fire-adaptive traits such as serotiny (retention of seeds on mature plants), smoke-induced germination and post-fire resprouting [15–17]. Fire regimes in these habitats can maintain open-canopy habitats, promoting diverse grass species favoured by sunlight [18] and, in turn, a variety of insects [19], land snails [20] and birds that thrive in fire-created niches [21]. Fire patterns can also contribute to maintaining a diversity of ecosystems [22]; for example, reduced fire frequency can transform open tropical savannas into closed forests [23,24].

Fire suppression in fire-prone areas has reduced fire frequency but led to fuel accumulation and more intense and severe wildfires [25]. Climate change is already amplifying fire danger, causing extensive wildfires in atypical seasons, elevations and vegetation types [26]. This rapid change is stressing species and ecosystems, even in fire-adapted regions [27]. Thus, understanding fire regime properties that preserve biodiversity under both natural and anthropogenic pressures is essential [28], while also prioritizing human health, safety and key ecosystem services like carbon storage or clean water supply [29].

Predicting how novel fire regimes influence biodiversity presents challenges [30]. Despite improvements in data collection and advances in modelling, the social and ecological complexities of fire-biodiversity relationships make it difficult to predict changes under new and unprecedented conditions [31]. Fire and biodiversity models are usually correlative, and rarely process-based, which complicates forecasting ecosystem dynamics and land use changes [32]. The stochastic nature of fire introduces further uncertainty. Integrative approaches, including testing the strategic use of prescribed burning, fuel management, wildfire suppression and other land management practices, require cutting-edge models and participatory approaches incorporating local knowledge and values.

This paper synthesizes scientific knowledge across taxa, ecosystems and continents to identify strategies for applying and managing fire in environments that differ from those experienced by biodiversity in the past. It begins by examining the main approaches to fire use, their biodiversity impacts and the challenges and limitations of these practices. It then introduces a decision-making framework to guide fire management for biodiversity conservation. This integrative approach to decision-making emphasizes the importance of clear management objectives, understanding biodiversity outcomes of alternative management strategies and iterative learning to address the challenges posed by novel fire regimes.

## 2. Fire use and practices

### (a) Broad approaches to fire use and management

One approach for maintaining biodiversity in fire-prone landscapes is to attempt to reinstate historical or reference fire regimes [33–36]. This can mean re-establishing fire as a natural ecological process. For instance, in North American longleaf pine ecosystems, a common objective is to revert to a pre-industrial fire regime, by considering as references the burning practices of Indigenous peoples and the role of natural ignitions [33,34]. Understanding the historic range and variation of fire regimes can broaden fire use possibilities and guide management towards more resilient landscapes [35]. However, re-establishing historical fire patterns in changing landscapes and climates without historical analogues is challenging [36].

Another widely used approach considers species and ecosystem characteristics to define suitable fire patterns [4]. This approach recognizes the context-dependence of burning strategies, varying with ecosystem type, historical fire regime, land management, conservation goals, and societal objectives and constraints [37].

Both of these approaches may involve the application of fire. While fire management spans a continuum of spatio-temporal scales, it is useful to consider two overarching types of planned burning: (i) broadcast burning, applied more uniformly over large areas (from hundreds to thousands of hectares) and emphasizing high coverage of burnt areas across the landscape; and (ii) patch-mosaic burning, conducted more heterogeneously at smaller scales (from less than a hectare to hundreds of hectares) and emphasizing the creation of patchiness and variation in burnt areas. Delineating burning strategies can help predict spatial and temporal fire patterns and their ecological effects. Broadcast burns are resource-intensive and come with risks of fire escaping its intended bounds and purpose. However, these large-scale burns can also be patchy depending on environmental variation (vegetation types, topography or fuel moisture) and ignition patterns, sometimes leaving 30–70% unburnt within a fire perimeter, as seen in examples from Australia [38], South Africa [39] and the western USA [40]. Patch-mosaic burning aligns more closely with traditional fire practices [41]. These two categories blur in real landscapes, where historical, traditional and current fire use often defies easy classification [42].

### (b) Biodiversity outcomes of historical and contemporary fire uses

Burning practices create different types of landscape mosaics [43] affecting biodiversity both positively and negatively (see examples in electronic supplementary material, table S1). A common aim is to promote particular vegetation types or species [3] by forming vegetation mosaics encompassing both younger and older successional patches [41]. These practices often involve low- to mixed-severity but frequent fires, set during moderate weather conditions to limit fire spread and intensity [44].

Fire is used to manage open habitats in various regions of the world. In British Columbia, Canada, a common goal is rangeland management, with wildlife benefits often a secondary consideration to production of livestock and pasture [45]. In the USA Great Plains, prescribed burning is used to restore the ecological functionality of grasslands by creating heterogeneous patches through a mix of fire and grazing practices [40]. However, annual burning still occurs over large areas, with some evidence that this results in uniform landscapes and reduced biodiversity [46]. In Southern Africa, a century of fire use in grassy ecosystems shows that modifying fire patterns may help to maintain herbaceous plant diversity and the animals that feed on them [47,48]. In Northern Australia’s tropical woodlands, shifting from late dry-season fires, which are often larger and uncontrolled, to early dry-season fires, which as typically smaller and patchier prescribed fires, may protect longer-unburnt habitats and enhance biodiversity by retaining important habitat structures [49,50]. Similarly, in the Brazilian Cerrado, some types of fire help maintain desirable structural and floristic components of savanna landscapes while reducing the occurrence of high-severity dry-season wildfires [51].

Planned fires are also used to manage woodland and forest habitats. In Scandinavian conifer forests, some types of prescribed fire promote more ‘natural’ conditions characterized by heterogeneous stand structures that favour pyrophilous and saproxylic organisms [52]. In many regions there is a need to reconcile wood production with conservation objectives. One way that this is being explored in the south eastern USA is through application of low-intensity fires in combination with silvicultural practices; evidence from stands of *Pinus palustris* indicates that this approach can maintain diverse understories of plants and fauna that benefit from a more open canopy [53]. Fire plays an important role in preserving heathland habitats of high conservation value in the UK and Europe [54]. However, in the UK, for example, planned burning in heathlands dominated by *Calluna vulgaris* primarily aims to maintain vegetation that supports the hunting of *Lagopus lagopus*. This has led to conflict between conservation objectives and the hunting objectives [55], with an ongoing challenge of maintaining fire intervals that promote *Calluna* and graminoids while preserving peat-forming mosses which are more sensitive to fire.

Although fire is also widely used to manage ecosystem services such as food and materials production [56], carbon maintenance [50] and clean water, the use of prescribed fires in many regions is primarily driven by the need to protect people from wildfires [37]. Hence, new frameworks are needed that consider biodiversity alongside other societal values.

### (c) Biodiversity-related issues and limitations of fire use

We identify four main challenges in applying fire for biodiversity conservation: (i) setting objectives for biodiversity conservation and planned burning, (ii) the complexity of fire-biodiversity relationships, (iii) uncertainty about past and future fire patterns, and (iv) creating the landscape types that meet biodiversity goals.

Setting ecological burn objectives is challenging due to the need to consider all species involved, not just a select few. A meta-analysis on the effects of prescribed burning effects on biodiversity found difficulties in detecting consistent relationships due to study heterogeneity and insufficient comparability and reporting across studies [57]. Limited information often leads to burning practices that prioritize more easily measured taxa, such as plants [37,58], while animals and their habitats are often neglected [58,59]. Moreover, growing evidence shows that using fire to benefit specific animals, like large savanna herbivores [37], could affect the overall biotic community. Thus, well-defined objectives should consider multiple taxa to achieve desired outcomes [36].

Fire patterns are complex and may threaten biodiversity in fire-adapted systems in a range of ways. In Australia, for example, fires that are too frequent can harm threatened vertebrates that require long-unburnt habitats [60–62]. At the same time, some threatened vertebrates are not getting enough of the ‘right’ kind of fire [63]. The timing of fires is also important: events outside the peak fire season can reduce flowering and alter seed chemistry of plants stimulated to flower by fire, such as *Doryanthes excelsa* from eastern Australia [64]. Broadcast burning for fuel reduction (approx. 5% of the landscape per year [65]) can lead to excessive juvenile vegetation, decreasing habitat for intermediate and mature seral species [66,67]. Therefore, complexity of fire–biodiversity relationships under a changing climate means that planned burning—whether it be broadcast burning or patch-mosaic burning—must carefully consider impacts on biodiversity at a range of temporal and spatial scales [68–71].

Another challenge is the uncertainty surrounding fire–biodiversity relationships under novel and emerging conditions. Relying solely on historical fire regimes as reference levels does not guarantee achieving desired ecological outcomes because many ecosystems now harbour new mixes of species, undergo more extreme climates and are subject to different human land-uses [33–35]. Invasion by alien plants, declining habitat quality, or loss of species associated with specific fire regimes are growing risks in many parts of the world [72]. Prescribed burning programmes aimed at achieving large-scale objectives, such as reduction in greenhouse gas emissions, may not necessarily produce local biodiversity co-benefits in fire-adapted ecosystems [50].

Lastly, achieving desirable fire patterns that promote conservation is difficult. While generating diverse or patchy fire patterns provide opportunities to conserve many species, a highly variable burning regime does not necessarily ensure increased biodiversity because desirable fire types, scale of burning and effectiveness are context-specific [73]. Generating adequate fire mosaics, including variation in patch size, connectivity [74] and time-since-fire distribution, is also difficult due to insufficient understanding of the ecological effects of different mosaics, contributing to management strategies that may not be fit for purpose [58]. A better understanding of context-dependence and key mechanisms underpinning fire–biota relationships can improve the creation of desirable forms of fire-driven variation, sometimes called pyrodiversity [75]. However, empirical studies indicate that pyrodiversity–biodiversity relationships are not straightforward [76], varying with climate [77], biota and ecosystems, and influenced by how pyrodiversity is defined and the spatio-temporal scale of analysis [78].

## 3. Best practices for biodiversity-enhancing fire management

**Figure 1.**
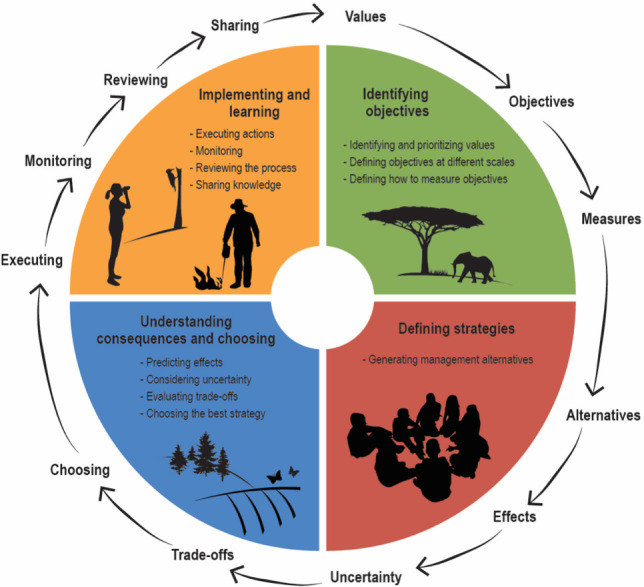
A decision-making framework to implement fire and biodiversity management under novel fire regimes.

The fast pace of fire-related changes [79], and increasingly novel conditions means that biodiversity management needs to be adaptive. One-size-fits-all approaches that overlook complexity and local context must be revised [80,81]. In the following sections, we describe a framework for enhancing decision-making practices in fire management for biodiversity conservation. We draw from several fields of research, including adaptive management [82,83], structured decision-making [84] and decision science [41]. The proposed framework (figure 1) encompasses the following steps: (i) specifying objectives and indicators for evaluating management alternatives; (ii) developing management alternatives to address the objectives; (iii) analysing potential consequences and considering trade-offs and uncertainties; and (iv) implementing strategies while monitoring their effectiveness, and sharing results to foster collaboration and new knowledge.

### (a) Identifying objectives

Effectively protecting biodiversity under novel fire regimes demands knowledge of ecosystems, as well as societal needs. Policymakers and practitioners often use a range of information to formulate fire management objectives, including spatial planning tools, such as habitat and species distribution maps, conservation plans, local knowledge about forest management and historical fire records. Indigenous and traditional fire knowledge, as well as Indigenous-led initiatives, are also increasingly recognized [1]. Engaging a wide range of community stakeholders provides valuable perspectives and helps clarify ambiguities for policymakers on the potential implications of their decisions [84]. It can also help to develop biodiversity objectives that are well-defined and measurable, improve their integration with other fire management objectives such as resource management, protection of lives and properties and supporting cultural practices [85], while also shifting towards allowing natural dynamics for self-organization [86].

Common objectives of ecological fire management include the generation of different types of pyrodiversity and fire mosaics [78], the preservation of threatened species and the restoration of ecosystem processes. More specifically, objectives can range from more precise goals [87], such as increasing food quality for a single species, like the mouflon (*Ovis orientalis*) in France [88], to broader goals, like creating open habitats for multiple species of invertebrates and birds [89,90].

**Figure 2.**
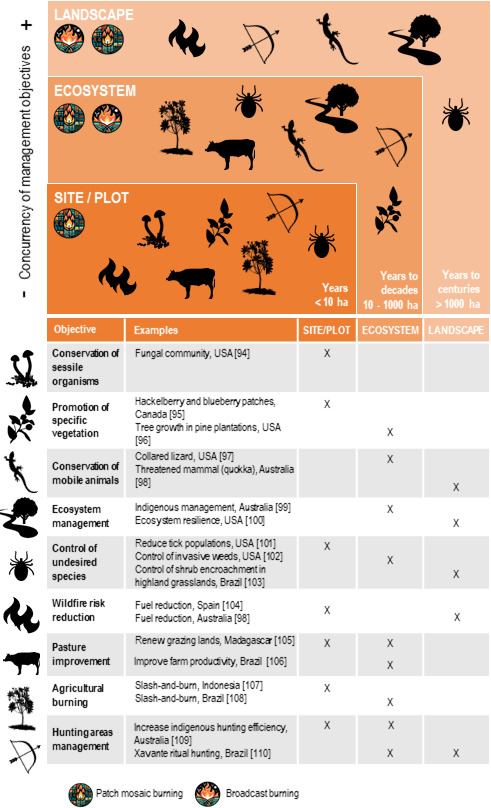
Examples of objectives of fire use aimed at improving biodiversity and other values at different spatial scales. In the attached table under the figure [91–107] provides more information.

A comprehensive understanding of trade-offs between multiple objectives is needed [41]. Broad scale or patchy burning can achieve multiple goals (figure 2), but landscape fire planning must balance diverse, scale-specific objectives. In the Pyrenees, for example, a strategic fire plan has the objectives of maintaining open rangelands and increasing habitat diversity, and actions to achieve these include smaller planned and larger unplanned fires [108]. After years of land abandonment in the region [109], this kind of prescribed burning is now helping to control shrub encroachment, recover pastoral value and reduce fire hazard. Although habitat improvement for birds of conservation concern is a positive side effect of management in the Pyrenees [56], fires that are too frequent can harm some aspects of biodiversity [110]. This highlights the need for a set of objectives that capture multiple biodiversity and ecosystem values.

Defining management objectives should involve identifying measurable attributes to assess goal fulfilment and adaptation [111,112]. New approaches are helping to do this. For example, fire severity mapping offers insights into vegetation recovery and biological legacies, crucial for understanding fire’s effects on biodiversity and ecosystem services. Emerging technologies, such as unoccupied aerial vehicles and acoustic recording devices, are also powerful tools for tracking post-fire changes in animal distributions [113]. Measurement of biodiversity and ecosystem processes enables ongoing monitoring, which facilitates adaptive management by identifying trends and opportunities to improve actions and strategies [114]. It also enhances fire management transparency and credibility by demonstrating the impact of different initiatives, supporting both conservation and socio-economic goals [115].

### (b) Defining strategies

Increased fire activity in many ecosystems experiencing more extreme climatic conditions intensifies the need for strategic intervention to promote biodiversity. A new approach is to use ‘adaptation menus’—collections of fire and climate adaptation strategies developed through science–management partnerships [116]. For example, climate change and increased fire activity are transforming montane and subalpine forests into grasslands in the Greater Yellowstone, USA [117,118]. In this region, fire suppression activities are more feasible in subalpine forests but what is on the ‘menu’ may differ depending on the vegetation type or region. For example, maintaining low fuel loads is essential in drier conifer forests to sustain frequent, low-severity fire regimes, as seen in Wyoming, USA [119].

Incorporating socio-economic aspects into fire management strategies involves acknowledging fire’s economic value, including burns for agriculture or traditional land-use [120]. Assessing unintended impacts of fire management on local economies, and ensuring interventions do not inadvertently harm people or assets, is also crucial. By considering socio-economic contexts and involving local stakeholders, fire management can move towards achieving ecological preservation while supporting community well-being and cultural values [121,122].

Globally, there is a wealth of Indigenous, traditional and local expertise in fire management and ecosystem stewardship [1,123,124]. In many fire-prone biomes, such as the Brazilian Cerrado, Indigenous fire practices enhance food availability for small vertebrates and arthropods, increasing species diversity [125]. In Australia, collaboration between Indigenous communities and land management agencies has positively impacted a range of ecological and social values [82,96,126]. Creating learning networks among scientific and non-scientific communities is one of many ways that people can come together to develop strategies that meet the challenges posed by novel fire regimes [127].

### (c) Understanding consequences

Analysis of whether or not fire management is working depends on the objectives and the spatial and temporal scales of the strategy (figure 2). This may include considering how a specific fire type affects a species’ distribution, population size and movement, or ecosystem structure and function, including metapopulation dynamics [6,127–129]. These effects should be considered at appropriate timescales, with fire initiating ecological changes from hours and days to decades or more [130]. Integrating this knowledge into the complexity and stochasticity of natural systems is necessary. A wide range of approaches are available to synthesize the consequences of fire management on biodiversity, including those that focus on a range of potential strategies and those more focused on a single best strategy or ‘optimal’ approach [32,120].

Identifying and quantifying uncertainty relating to the consequences of fire management is important. Under novel fire regimes, in the physiology, phenology, composition and structure of plant communities is likely to be modified [131]. Climate and fire regime changes may render some data and knowledge inadequate for new conditions [132]. To address this, a useful approach for building on ‘best practice’ knowledge is to integrate species vulnerability assessments, that to date are largely focused on climate change, with new empirical observations [133]. There are exciting opportunities to develop this approach to fire regimes and the resulting shifts in biodiversity [112]. This challenge is particularly evident in ecosystems like South African savannas, where low-intensity grass fires can transition into high-intensity 'firestorms' under specific conditions, exacerbated by climate change [134]. Such extreme conditions alter ecosystem dynamics and complicate the implementation of prescribed burns [135]. Therefore, decision-makers must consider a range of values—including cultural, social and economic factors—when developing strategies to address these uncertainties [136,137].

Once the consequences of management alternatives are estimated, and uncertainties accounted for appropriately, clarifying trade-offs between different strategies is a useful next step [84]. This involves examining how different objectives, and performance measures, are likely to change under alternative fire management strategies [138]. Fire management strategies can harm biodiversity [41]. So, recognizing that there are trade-offs is crucial for informed decisions about biodiversity conservation in the context of dealing with other values such as human health and built assets (Box 1).

Box 1. **Examples of trade-offs in fire use and possible ways to navigate them.**

*Reduce wildfire risks to people versus preserve natural fire regimes*: fire suppression to protect human communities can disrupt historical fire regimes [139]. Taking a long-term perspective may help to balance immediate protection of lives and property with ecosystem health.

*Improve grazing quality versus protect native biodiversity*: fire can benefit grazing animals by removing shrubs but may also spread invasive plants [140]. Understanding this risk, spatial planning may help avoid burning in environments sensitive to plant invasion and grazing animals.

*Wildfire prevention versus biodiversity conservation*: prescribed burning for hazard reduction can negatively impact biodiversity. A risk-based framework provides opportunities to carefully assess the outcomes of prescribed burning on multiple values while considering critical uncertainties [72].

*Fuel management versus tree species composition*: prescribed burning in *Pinus yunnanensis* forests benefits some understorey *Quercus* species but harms others taxa [141]. Better knowledge of plant life histories and regeneration capacities aids conservation and fuel management goals.

*Carbon storage versus biodiversity conservation*: managing forests for *Leuconotopicus borealis* increases biodiversity but decreases carbon storage potential [142]. Trial prescribed burning and thinning to balance species conservation and carbon sequestration objectives.

*Traditional pasture management versus natural area protection*: restricting fire in a protected area conflicts with traditional grassland renewal by fire, and may negatively affect biodiversity [143]. Understanding the consequences of fire through experimentation with local communities can help to develop conservation objectives, while meeting cultural and economic goals.

### (d) Implementing and learning

In the context of novel fire regimes, effective information exchange, planning and preparation are essential for implementing strategies successfully. However, administrative, social, legal, logistic, budgetary and weather-related issues can delay or cancel burning programmes. Flexibility—openness to dialogue, adapting schedules, modifying techniques or redistributing resources—helps overcome these obstacles. For example, with an estimated 17% reduction in the window for prescribed burning in the western United States under 2°C of global warming, adjusting burning periods will be necessary [144], though identifying the optimal window requires tools not always available to managers.

Monitoring should have clear objectives and efficient methods for comparing attributes across fire management scenarios over time. Methods must balance effort (time, personnel, logistics and budget) with quality data. Monitoring, combined with mapping, occurs in phases: before, during and after the burn. Pre-burn data informs the burn plan and establishes a baseline [145]. Data collected during the burn helps refine operations [146]. Immediate fire effects—from structural changes to organism mortality—may not be fully apparent initially [147], but monitoring over weeks to years allows a comprehensive assessment of ecological resilience and management effectiveness. Long-term monitoring is required to assess ecosystem resilience, including vegetation and wildlife recovery [110].

Observational, experimental and modelling approaches are invaluable for predicting outcomes of prescribed fires and integrating this knowledge into planning. BACI (before-after-control-impact) designs are useful for assessing ecological effects of fire [148,149], but they are logistically demanding, expensive and may not yield rapid results [150]. Alternatively, before-after-only or control-impact designs are also useful, provided there is adequate sample size and spatio-temporal replication, and treatment interspersion and synchronic sampling of burnt and unburnt sites [145].

Reviewing progress towards goals is essential. It involves assessing objective achievement, unexpected results, and stakeholder satisfaction, prompting evaluation. Identifying areas for improvement or incorporating new information should lead to adjustments at any stage (figure 1). This process is particularly crucial as the environment rapidly change and uncertainty increases [82].

Effective knowledge dissemination must prioritize stakeholder inclusivity, ensuring their contributions are recognized and they are well-informed about decision implications. This transparency fosters ownership and supports future initiatives [151]. Moreover, transferring new knowledge to policymakers and stakeholders helps reduce ambiguities in future decision-making.

## 4. Fire management for biodiversity: concluding remarks

Effective fire management under novel fire regimes requires an evidence-based approach. Biodiversity-focused fire management must recognize fire’s ecological role, adapting strategies to mimic natural or historical fire regimes while accounting for novel conditions driven by rapid environmental changes. This demands clear objectives, long-term ecological knowledge and robust climate predictions. Evidence-based management, supported by diverse research methods and long-term studies, is essential to assess trade-offs and address knowledge gaps. Monitoring ensures alignment with conservation goals, with feedback mechanisms enabling ongoing adjustments. Effective management integrates fire activities with broader land management and acknowledges fire’s ecological and cultural significance.

To ensure fire management for biodiversity conservation can deal with emerging conditions, we suggest the following ways forward: (i) consider both social and ecological factors that influence fire and biodiversity and how they are valued; (ii) establish long-term biodiversity monitoring in fire-affected ecosystems making use of new remote sensing technologies (like UAVs and LiDAR), opportunities for on-ground and real-time observations of ecosystems, to refine strategies; (iii) prioritize cross-disciplinary collaboration that fosters knowledge among and between local communities, fire practitioners, conservation managers and scientists; and (iv) implement iterative testing of fire management actions, supported by adaptive management and experimentation (such as pilot projects), and continual learning. This integrated approach to learning about fire and biodiversity will help to navigate the complexities of novel fire regimes and preserve biodiversity in a fast-changing world.

## Data Availability

Supplementary material is available online [152].
